# Behavioral Impact of Unisensory and Multisensory Audio-Tactile Events: Pros and Cons for Interlimb Coordination in Juggling

**DOI:** 10.1371/journal.pone.0032308

**Published:** 2012-02-27

**Authors:** Gregory Zelic, Denis Mottet, Julien Lagarde

**Affiliations:** Movement To Health, EuroMov, Montpellier 1 University, Montpellier, France; The University of Western Ontario, Canada

## Abstract

Recent behavioral neuroscience research revealed that elementary reactive behavior can be improved in the case of cross-modal sensory interactions thanks to underlying multisensory integration mechanisms. Can this benefit be generalized to an ongoing coordination of movements under severe physical constraints? We choose a juggling task to examine this question. A central issue well-known in juggling lies in establishing and maintaining a specific temporal coordination among balls, hands, eyes and posture. Here, we tested whether providing additional timing information about the balls and hands motions by using external sound and tactile periodic stimulations, the later presented at the wrists, improved the behavior of jugglers. One specific combination of auditory and tactile metronome led to a decrease of the spatiotemporal variability of the juggler's performance: a simple sound associated to left and right tactile cues presented antiphase to each other, which corresponded to the temporal pattern of hands movement in the juggling task. A contrario, no improvements were obtained in the case of other auditory and tactile combinations. We even found a degraded performance when tactile events were presented alone. The nervous system thus appears able to integrate in efficient way environmental information brought by different sensory modalities, but only if the information specified matches specific features of the coordination pattern. We discuss the possible implications of these results for the understanding of the neuronal integration process implied in audio-tactile interaction in the context of complex voluntary movement, and considering the well-known gating effect of movement on vibrotactile perception.

## Introduction

In everyday natural situations the combination or integration of multiple senses is essential for an adapted goal directed behavior [1]–[8]. The efficiency of multimodal integration is demonstrated by the improvement of detection, reaction, and discrimination [9]–[15]. Recently it was found that an added sound can interfere with the opponent's perception of the ball in tennis, showing that multisensory integration may play a strategic role also in more ecological settings [16]. Yet little is known about how cross-modal environments can contribute to the ongoing coordination of limbs in complex tasks [17]–[19]. In this study we examine multisensory processes defined neither by detection or discrimination, nor by behavioral reactions after the presentation of cross-modal stimuli, but involved when perception and action come together in ongoing coordination. The coordination of movements has a pervasive functional role in elementary behaviors [20]–[23] (e.g. grasping, reaching, pointing, upright standing, walking, chewing, speech production, to name a few), in daily actions (see [24] for an illustration), but also at the work place, in performing music and arts, or in sports [25]–[27]. Such coordination typically involves multiple joints, and requires dynamic and reciprocal information exchanges between brain, body and the environment [22], [23], [28]–[31]. Though intrinsically multisensory (i.e. combining vision, audition, touch, and proprioception), interlimb coordination is in many cases very dependent upon the use of vision. Here we are interested in the advantage audio-tactile stimuli, which leave the optical array invariant, may provide to specify, guide, and enhance coordination. Despite the recent increase of interest for audio-tactile multimodal integration its impact on behavior is still poorly documented (for a review see [32]).

It is well known that detection of tactile events is reduced during the execution of movements, a gating effect [33]–[36], which varies during the time course of movement [37]. If the information provided by external tactile events can hardly be detected when movements are produced, then one may predict that audio-tactile stimuli are not likely to improve coordination skills. However in the present study the tactile detection was not achieved without a functional relation to the movement produced; rather the stimuli endorsed the role of carrying over relevant information to enhance an ongoing coordination. Moreover vibrotactile stimuli have been successfully used to direct attention in driving tasks [38]. Therefore, on the basis of these previous studies, the efficiency of audio-tactile events to drive the coordination of movements remains an open question.

We selected the so-called three balls cascade juggling trick, for which the tactile suppression phenomena has recently being demonstrated [39], as test case in the present study. The three balls cascade is among the simplest and the most studied juggling tricks [40]–[43] and will be denoted juggling in the present paper. Juggling represents a very challenging and high-dimensional coordination problem. On the perception side, vision is cardinal to dynamically couple the hands to the balls pattern [40], [44], [45] and involves significant neural plasticity [46], [47]. On the movement side, juggling requires the patterning of precise and fast hand movements [41], [42]: the juggling pattern is periodic and characterized by an invariant relative timing, where the throwing action parameterizes the parabolic trajectories of the ball [43]. In terms of coordination, juggling is mainly characterized by a 1∶3 frequency ratio between the hand movement and a ball cycle [41], [42]. This notably entails forming and maintaining multi-frequency relations among movements of hands, eyes and posture [40], [43]–[45], [48]–[50]. Proficiency in juggling is readily identified by co-variations among the trajectories of the hands and the limbs combined with a decrease in variability of balls trajectories in time and space [42], [51], [52], which contributed to depict learning to juggle as the formation of a “spatial clock” [43].

What type of external periodic stimulations could improve the performance at juggling?

Sensory stimulation can increase the robustness of elementary coordination against internal biological noise or external perturbation [23], [53], [54]. This increase in robustness was accompanied by a decrease in variability of limbs trajectories, a phenomenon referred to as “anchoring”, which indicates higher stability of the coordination pattern [55]–[58]. In the case of interlimb coordination, the stabilization effect was even more pronounced when the frequency of the stimuli was twice the frequency of the movement of the limbs [59]. Periodic stimuli carrying information about the tempo provide global frequency information, but also local relative timing information specifically defined by phase difference [54], [60]. As juggling is a rhythmical skill, such external timing information should be beneficial to the juggler. However, a simple auditory metronome failed to stabilize juggling [61], which indicates that the multi-frequency nature of the juggling calls for more than a mere metronome.

To provide the juggler with two metronomes without overloading vision, we assumed that non-visual cross-modal pairings matching the multi-frequency nature of juggling would be the best stimuli to improve the stability of the coordination. Accordingly, we focused our study on audio- tactile parings. Previously important principles underlying the combination of sensory cues from different modalities have been discovered, notably that integration takes place according to a statistical optimal rule [62]. However to the best of our knowledge, only anecdotal evidence of an advantage of cross-modal stimuli over uni-modal parings was provided for this class of multi-frequency coordination in a bimanual task [63]. This corresponds to a case where cross- modal pairing may be adequate because the task requires segregation to move the hands at two distinct frequencies.

In the present study, our prediction that one can stabilize the juggling coordination with adequate external sensory stimulations was tested using periodic audio-tactile stimuli, respectively presented at the ears and at the wrists, and providing specific tempo. Because balls and hands frequencies are core to the multiple component coordination in juggling, we chose to associate each sensory modality to one of these main components. The frequencies of the tactile metronome and of the auditory metronome were thus scaled to match, respectively, the tempo of the hands and the tempo of the balls. In one condition, the metronome frequencies were multiplied by two to examine the generalization of the parametric stabilization to an audio-tactile pairing [59], [64] (see also [65]). Finally, in one audio-tactile condition, the tactile stimuli were presented alternatively at each wrist, so to match the antiphase movement of the two hands in juggling [23], [53], [54].

## Methods

### Participants

Seven right-handed students (five males, mean = 23.5 yr, SD = 2.5 yr) from the Montpellier 1 University participated in this study. Each participant signed an informed consent form approved by the Institutional Review Board (IRB) of the Montpellier 1 University (UFR STAPS).

Because of the very low natural variability of expert jugglers [66] and because novices cannot juggle in a sustained fashion, we ran a preliminary test to identify intermediate-level jugglers: participants performed a three-ball cascade, while keeping feet inside a 2 meters circle. They were deemed intermediate-level jugglers if they could succeed for at least 20 seconds, but failed before 60 seconds. Seven volunteers were selected out of thirteen tested.

### Apparatus

Data were collected using a Vicon 3D motion recording system at a 100-Hz sampling rate. The system was calibrated according to the manufacturer's instructions prior to data collection. Two reflective markers were taped on the dorsal head of the 3^rd^ metacarpal, to record the left and right hand movements. The three balls were covered with reflective tape and defined as markers, to record the movements of the balls.

Thanks to additional markers fixed on each shoulder, we had able to follow the rotation and translation movement of the subject in the environment during the task. To homogenize the set of trials, we realized a projection of the data, first recorded in the lab frame, into a new reference frame relative to the subject (center at the top of the sternum – O , with antero-posterior – 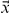, transversal – 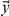, vertical axes – 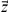), fixed in time, and defined as the mean position of the participant on the trial.

The vibrotactile metronome consisted in 80 ms square wave pulses (vibration carrier frequency: 100 Hz). The stimulation was delivered with a DC motor (weight: 42 g) strapped on the ventral part of each wrist. Preliminary tests showed that the time to reach 100 Hz was less than 5 ms.

The auditory metronome consisted in 80 ms square wave pulses (tone carrier frequency: 300 Hz) delivered to headphones fixed on top of the auricles. Headphones also played white noise in order to isolate the subject from the noise emitted by the vibrotactile stimulation.

### Conditions

We wanted to compare the effect of metronomes that differed in modality (tactile, auditory or audio-tactile), frequency (simple or double) and, in the case of simple frequency metronomes, in the phasing of the stimuli (in-phase or anti-phase).

Running the full crossing of the three factors in a repeated measure design would necessitate multiple sessions per participant. As a consequence, we selected the most interesting metronome conditions. Because metronomes that beat twice per movement generally provide a better guidance [59], we selected such a double frequency metronome in the three modalities (Multi. Doub., Tact. Doub., Audio. Doub.). Then, to reproduce the natural relative phase between the hands movement in juggling [23], [53], [54], [67], we added the simple audio-tactile metronome with anti-phase tactile stimuli on each wrist (Multi. Anti.) and its in-phase counterpart (Multi. Simp.). Finally, we included a baseline control condition with no metronomes, to get a total of 6 experiment conditions (Table 1).

**Table 1:** Experimental conditions and characteristics of the metronomes.

Sensory Modality	Structure	Auditory frequency	Tactile frequency (and phasing)	Label
Audio-tactile	Simple	Ωball	Ωhand (Π = 0)	Multi. Simp.
Audio-tactile	Simple	Ωball	Ωhand (Π = π)	Multi. Anti.
Audio-tactile	Double	2*Ωball	2*Ωhand (Π = 0)	Multi. Doub.
Tactile	Double	-	2*Ωhand (Π = 0)	Tact. Doub.
Audio	Double	2*Ωball	-	Audio. Doub.
-	-	-	-	Control

The sensory modalities (multimodal – solid line frame – vs. tactile or auditory unimodal) and the metronome parameters have been manipulated. We distinguished simple metronome from double metronome (dashed frame) for which the tactile and auditory metronome have been set equal to or twice the hands and balls frequency respectively. In one of the simple, multimodal metronome, the vibrotactile stimuli were presented in antiphase at the wrists (Multi. Anti, Π = π). Otherwise Π = 0 indicates that the vibrotactile stimuli were presented simultaneously.

**Figure 1:**
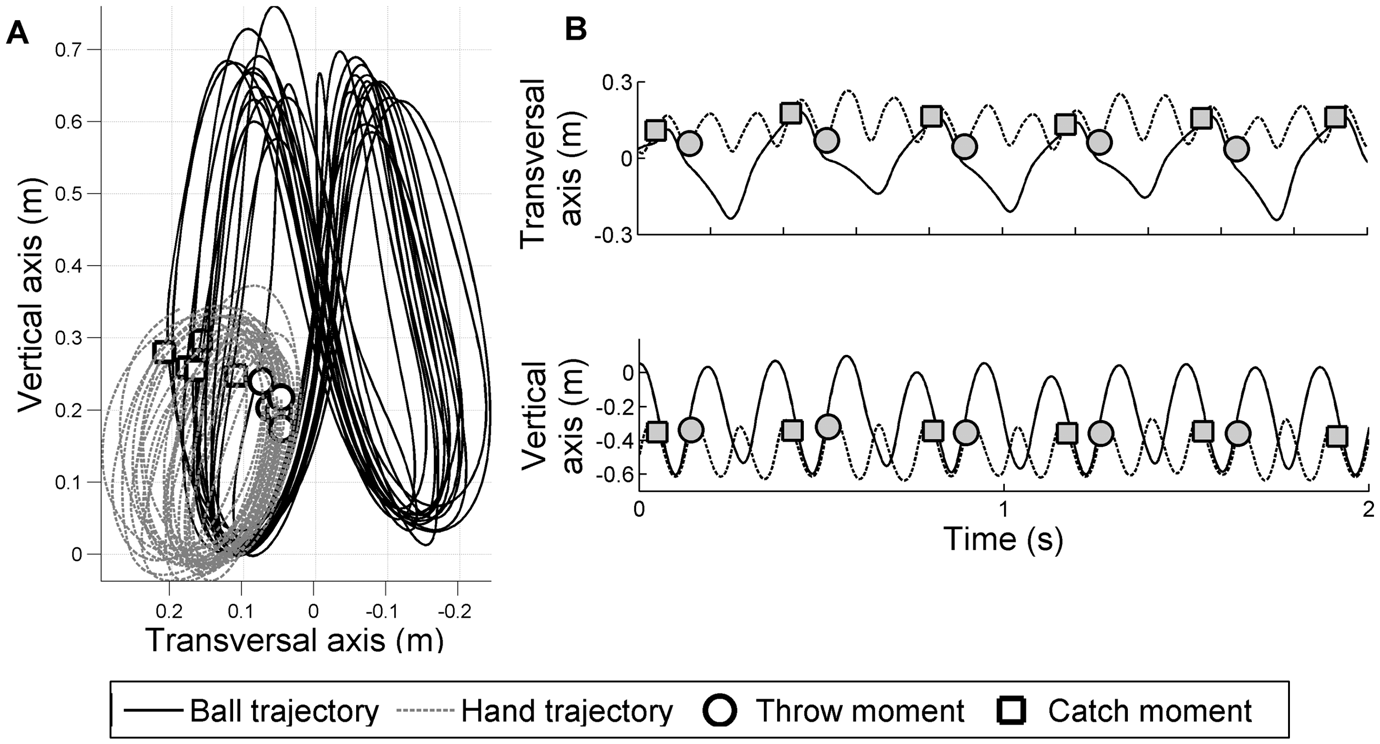
Representative juggling behavior in the frontal plane 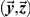. (A) Motion of balls (solid line) and left hand movement (dashed line) in the frontal plane are presented with the localization of throws and catches (respectively circles and squares). Please note that throws and catches points and balls trajectories are not exactly coincident because the passive marker was placed on the back of the hand. (B) Representative time series of position of one ball and of position of the left hand are respectively represented in solid and in dashed lines.

More precisely, because the juggling is the assembly of two frequencies within a single task [41], [48], [52], we set up the tactile metronome to the hand frequency (Ωhand) and the auditory metronome to the ball frequency (Ωball). Hand frequency was the inverse of the average period of time between two consecutive throws by the same hand. Ball frequency was defined as one-third of hand frequency, because one hand takes care of three balls in the selected juggling pattern [41]. In the multimodal conditions, the phasing between the audio and the tactile stimuli corresponded to the average phasing between the motion of one ball and the motion of one hand along the vertical axis, that is, the two stimuli were set with identical initial phases (see Figure 1, panel B).

### Procedure

We used a repeated measures design, where each participant had to juggle in each one of the 6 metronome conditions. The order of the metronome conditions was randomized across participants. In each metronome condition, participants had to perform a bloc of 5 trials.

The experimental session started with a preliminary trial to record the preferred juggling frequency of each participant. Then, during 10 minutes, the participant was familiarized with the 6 metronome conditions. After this familiarization, the participant performed a block of 5 trials in each one of the 6 metronome conditions, for a total of 30 experimental trials.

No instructions to synchronize with the stimuli were given, and participants were instructed to start juggling after the metronome started.

The task was to juggle as regularly as possible for the duration of a trial: 20 to 30 s (in some conditions, two jugglers were only able to complete 3 trials lasting more than 20 sec.).

For each participant individually, the frequencies of the metronomes were set at her/his preferred hands frequency (or twice the preferred frequency in the Doub. conditions).

### Data processing and analysis

The marker data were dual passed through a second-order Butterworth filter (fourth-order) with a low-pass cutoff frequency of 8 Hz prior to further data processing.

From the vertical velocity profiles of balls, we identified the moments of throw and catch: a positive velocity peak denotes a throw, and a negative velocity peak denotes a catch.

We then used the average period of time between two consecutive throws by the same hand to compute the hand frequency (Ωhand).

**Figure 2:**
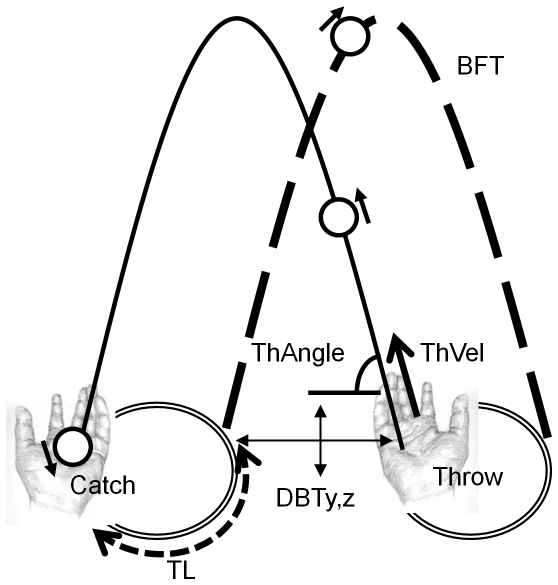
Juggling pattern in the frontal plane 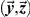. Hand trajectories are represented with a double line, and ball trajectories with a single line. Ball trajectories are presented during ball flight time (BFT) : the trajectory of a ball thrown by the left hand is drawn with a solid line, the trajectory of a ball thrown by the right hand is drawn with a dashed line (see Table 2 for variables details).

We addressed the organization of the juggling by analyzing the variability of the juggling pattern among the metronome conditions. Variability was computed as the within-trial standard-deviation of the variables of interest, using directional statistics for angular values [68]. The variables, listed in Table 2 and schematized in Figure 2, measured the performance in three aspects of variability that are key to sustained juggling: timing, bimanual coordination, and throw.

**Table 2:** Dependant variables.

Label	Name	Definition	Computation
BFT	Ball flight time	Time duration between the throw of a ball and its consecutive catch	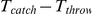
K	K-ratio	Proportion of time with the hand loaded (TL - i.e., with ball in hand) in a juggling cycle	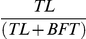 with 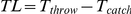
DBT	Distance between throws	Euclidean distance between two consecutive points of throws in the vertical plane	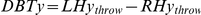 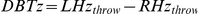
ThVel	Throw velocity	Norm of the velocity vector at time of throw in the plane of ball flight	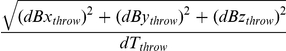
ThAngle	Throw angle	Angle of the velocity vector at time of throw in the plane of ball flight	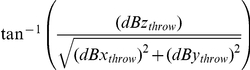
FSI	Frequency synchronization index	Difference between the hand frequency and the metronome frequency	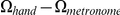
PSI	Phase synchronization index	Dispersion of the relative phase	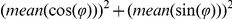
DT	Dwell time	Percentage of phase locking episodes	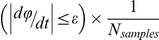 with ɛ a limit value and Nsamples the number of values in a trial

We analyzed the within-trial standard-deviation of the dependant variables listed in this table. The first two variables (BTF, K) are used to assess the juggling performance in time. The third variable (DBT) addresses the spatial aspects of the coordination between hands in the transversal axis (DBTy) and in the vertical axis (DBTz). Two variables (ThVel, ThAngle) focus on the throwing, which is key to sustained juggling. Note that, though the juggling was performed in 3D space, ThAngle refers to the elevation angle in the plane of ball flight. The three last variables (FSI, PSI, DT) were used to assess the synchronization of the participant with the metronome. Hand frequency (Ωhand) was the inverse of the average period of time between two consecutive throws by the same hand. The metronome frequency (Ωmetronome) was the inverse of the period of the metronome.

In the equations of the computation column : T is the time ; Hx, Hy, Hz are the coordinates of the hand ; Bx, By, Bz are the coordinates of the ball ; indices specify the juggling events (i.e., catch, throw) ; d indicates the differential, so that *dQ_throw_* is the differential of Q at time of throw ; L/R refers to the left/right hand ; ϕ is the relative phase between hand position along the vertical axis and the metronome.

First, because the timing of balls events seems to be the focus of control in intermediate and expert jugglers [52], we examined the within-trial variability of ball flight duration. The ball flight time (BFT) is the period between a throw and the consecutive catch by the other hand (Table 2 and Figure 2). Variability in BFT is here used as a global indicator of the stability of the coordination involved to sustain juggling. We also investigated the within-trial variability of the time spend with a ball in hand. The so-called K-ratio (K) is the percentage of a cycle duration with the ball in hand (Table 2). The K-ratio is a key variable in the temporal structure of the juggling [43], [55], [61].

Second, we addressed the organization of the juggling in space via the within-trial variability of the distance between two consecutive throws in the vertical plane (DBTz, see Table 2). Because two consecutive throws involve the two hands, these variables render a global measure of the between-hand coordination in space.

Finally, we captured the organization of the throwing behavior via the within-trial variability of the tossing: The tossing angle and velocity fully determines the trajectories of a ball (ThAngle and ThVel, see Table 2 and Figure 2).

### Synchronization to metronome

Synchronization of the hands to the metronome should result in systematic frequency relations (i.e., frequency locking) and, if the coupling is strong enough, in systematic phase relations (i.e., phase locking) between the hands and the metronome [69].

Frequency locking was easy to assess from a frequency synchronization index (FSI) computed from the difference between the observed hand frequency and the frequency of the metronome (see Table 2).

Phase locking necessitated a more sophisticated approach. For each hand, the instantaneous phase was estimated using the Hilbert's transform of the vertical positions time series [70]. The periodic variability arising from the specific coordinates used to extract the instantaneous phase [71], [72] was reduced by the method proposed by Kralemann et al. [73]. The relative phase between a hand and the metronome was obtained by probing the value of the hand's phase at times of stimuli onsets, which is tantamount to using the metronome as a stroboscope to read the hand's phase time-series. To measure the strength of the phase synchronization, we computed a synchronization index PSI (see [74], for a theoretical presentation). The value of PSI is zero in the absence of synchronization, corresponding to a uniform distribution of relative phase. The value of PSI is one for perfect synchronization, corresponding to a distribution concentrated around a single peak [75]. This phase synchronization index is formally analog to the dispersion of the relative phase distribution [68] and was calculated using the following formulae: 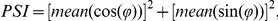
where PSI is the phase synchronization index and φ is the relative phase between the position of the hand along the vertical axis and the metronome.

Furthermore, we calculated the dwell time of the relative phase ([23], chap.4). The dwell time was introduced in the case of intermittent phase locking [58], and can be used to assess tendencies toward phase synchronization [76]. We calculated the dwell time from the time series of the slope of the relative phase (i.e., relative phase velocity). A slope close to zero corresponds to an episode of stationary phase locking [19]. A tendency toward synchrony is revealed by alternating episodes of large and small values of the slope, which indicates episodes of change and dwell in relative phase. The dwell time is the importance of the small slope episodes expressed as a percentage of the total length of the trial: the larger the dwell time the stronger the tendency to synchronize.

Operationally, to minimize the effect of noise after differenciation of the relative phase, we smoothed the time series of slope with a 4-points moving-average before calculating the proportion of time spent in small-slope episodes (i.e., episodes where the absolute value of relative phase velocity was lower than a limit value). We tried four different limit values for a small-slope episode (0.1, 0.15, 0.2, and 0.25 rd/s). The dwell time (DT) is the time spent in small-slope episodes divided by the total duration of the trial and multiplied by 100 to get a percentage.

### Statistical analysis

To assess the significance of the effects of the metronomes on the juggling performance, we analyzed the variability of the juggling and, in a second step, focused on the synchronization to the metronomes, in relation to the juggling performance.

All analyses of variance were complemented with Tukey HSD tests for post-hoc mean comparisons, and we reported the raw F values and degrees of freedom, but *p* values after Huynh-Feldt correction for non spherical variance.

In all statistical analyses, the significance level was set at .05.

#### Effect of the metronomes on the variability of juggling behavior

Participants clearly differed in their level of variability in the control condition, which corresponded to differences in their juggling experience, despite the inclusion test and criteria. Accordingly, to improve the sensibility and reliability of our analysis to distinguish the five metronomes conditions, we subtracted for each subject his/her mean variability measured in the control condition to the variability measured in the metronomes conditions, and this for each given variable. Thus in this part of the analysis all variables were baseline corrected.

To examine the effect the 5 metronome conditions on the juggling performance, we ran a global analysis of variance with repeated measures (ANOVA). Moreover, in order to test for significance against the baseline level, we compared the juggling performance under each metronome condition against the hypothetical mean of zero, the latter corresponding to the baseline level of variability, using a one sample *t*-test. As we emphasized the sensitivity of the analysis, we did not use a Bonferroni-Sidak correction [77], [78] and kept the *p* value threshold set at .05.

#### Entrainment to the metronomes

To globally address the issue of synchronization, we analyzed the timing of the juggler's hand relative to the metronome beats, using an ANOVA with repeated measures, as previously.

In a last step, being aware that mean frequency differences between movements and the metronome presented (FSI) varied between subjects, we further examined the phase synchronisation to the metronomes in each participant individually. We ran a within-participant statistical analysis using a surrogate approach to distinguish true phase synchronization from values obtained by chance. One type of synchronization, a weak synchronization, can indeed be expressed by a correspondence between the mean frequencies while phase synchronization is absent [22], [69]. For instance consider the fact that oscillatory movement may comprise, except for perfectly harmonic movement, alternating slow and fast evolution within a period. In such a case periodic stimuli onsets would necessarily happen more often during the slow parts, introducing a concentration of the relative phase distribution, directly increasing the synchronization index and dwell time. The phase synchronization variables would differ from zero but this won't indicate an underlying sensori-motor phase synchronization process. In this case rejecting the presence of phase synchronization requires a specific approach. We then tested the hypothesis of phase synchronisation by comparing the observed distributions of the synchronization index and dwell time to surrogates distributions of the same quantities. The surrogates were generated under the null hypothesis of values obtained by chance in the absence of coupling to the metronome. To this end we computed a relative phase between the movements recorded in the control condition and a metronome generated from those used in the other conditions. Moreover the time between the start of the movement and the first stimulus onset was randomly drawn from a Gaussian distribution, so as to get for the initial time shift a duration between 0 and the actual period of the metronome used for a given subject. By taking 1000 random initial period in different runs we computed for each individual 1000 phase synchronization indexes and dwell times (see appendix S1).

Because larger values indicate better synchronization in the surrogate distributions obtained for each participant, we took the 95 percentiles as the threshold value to get a p value of 0.05 for significant synchronization [79].

Finally, we analyzed the correlation between the synchronization indexes and the variability of the juggling pattern, with the prediction that better synchronization to the metronome would render lower variability in the juggling pattern.

## Results

Figure 1 A–B presents a representative sample of the recorded data, which clearly demonstrates the variability in hands movements, onsets of throws and catches, and in the ball's trajectories.

### Throwing movement

The first step in the analysis of the data was to examine whether the temporal information carried out by the external metronome affected the throwing action. The ANOVA applied on the variability of the initial angle at throws (ThAngle) was inconclusive.

**Figure 3:**
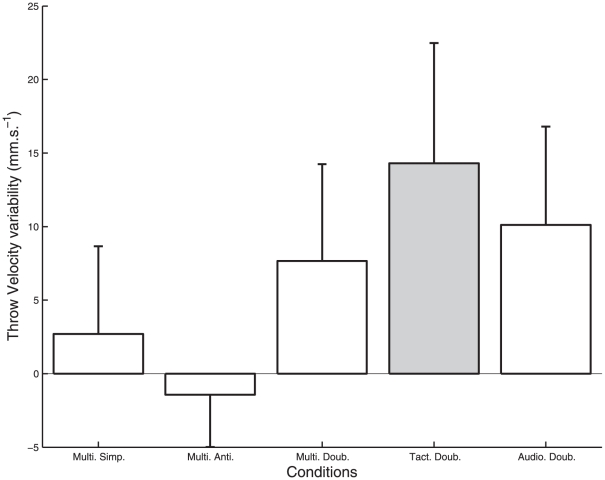
Variability of throw velocity (ThVel) for each metronome condition. The average variability for each individual in the control condition defined the individual baseline variability, which was subtracted to the individual's average variability in each metronome condition. Thus, the zero corresponds to the baseline variability without metronome. Negative values indicates smaller variability than in the control condition. Error bars represent inter-participant standard deviation. The grey bar indicates a significant increase in the variability of throw velocity in the Tact. Doub. metronome condition.

The ANOVA on the variability of the velocity at throws (ThVel) indicated an effect of the metronome condition (Figure 3; F_4, 24_ = 3.03, p = .037). It especially showed that the velocity variability with the antiphase audio-tactile metronome (Multi. Anti.) was smaller than the variability obtained with the unimodal tactile metronome (Tact. Doub.).

In addition the t-test comparison showed a variability of the velocity at throws in the unimodal tactile condition (Tact. Doub.) significantly larger than zero (t_30_ = 2.13, p = .042).

### Temporal organization of the juggling coordination

The ANOVA applied on the averages of variability of the K-ratio was inconclusive.

**Figure 4:**
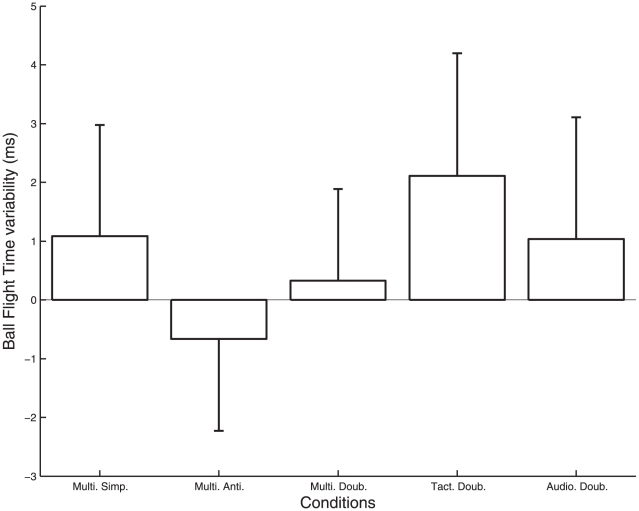
Variability of ball flight time (BFT) for each metronome condition. Zero corresponds to the baseline variability without metronome, and the error bars represent inter-participant standard deviation. The lack of grey bars indicate no significant difference from baseline variability: the metronomes did not influence significantly the BFT.

The ANOVA on the variability of the balls flight time (BFT) revealed a significant effect of the metronome condition (Figure 4; F_4, 24_ = 3.60, p = .030). It evidenced a smaller variability of the BFT in the antiphase audio-tactile condition (Multi. Anti.) than in the unimodal tactile condition (Tact. Doub.).

### Spatial variability of the juggling coordination

**Figure 5:**
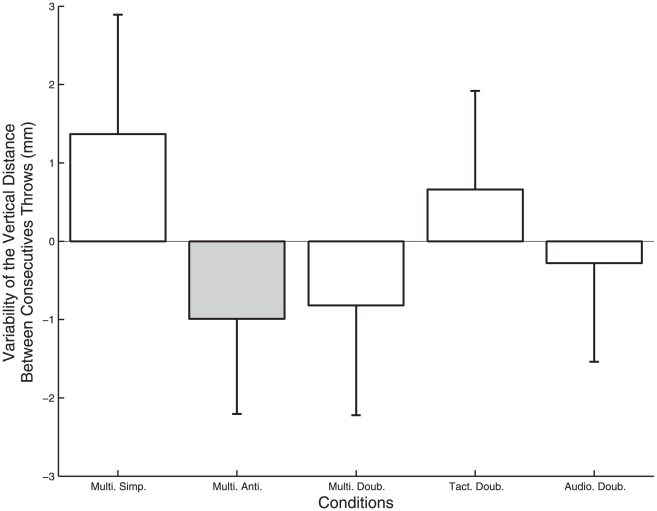
Variability of the vertical distance between consecutives throws (DBTz) for each metronome condition. Zero corresponds to the baseline variability without metronome, and the error bars represent inter-participant standard deviation. The grey bar indicates a significant decrease in the variability of the vertical distance between consecutives throws in the Multi. Anti. metronome condition.

The ANOVA on the variability of the vertical distance between two consecutives throws (DBTz) also showed a significant effect of the metronome condition (Figure 5; F_4, 24_ = 3.62, p = .019). The antiphase audio-tactile condition (Multi. Anti.) decreased the DBTz variability when compared to the in-phase audio-tactile condition (Multi. Simp.). In addition, let us remark that post hoc comparisons suggested, with a probability very close to the threshold for significance (p = .051), that this variability was smaller with the audio-tactile double metronome (Multi. Doub.) than with the audio-tactile simple metronome (Multi. Simp.).

Finally, t-tests showed that the variability of DBTz reached in the antiphase audio-tactile condition (Multi. Anti.) was significantly smaller than the zero baseline level corresponding to the control condition (t_30_ = −2.24, p = .033).

### Entrainment to the metronome

The ANOVA on the frequency synchronization index (FSI) was not conclusive.

In addition, neither the ANOVA on the phase synchronization index (PSI) nor the ANOVA on the dwell times (DT) showed a significant effect of the metronome conditions.

However, as the differences between the actual average hand frequency and the metronome frequency varied between participants, this may have overridden the detection of differences in phase synchronization between metronomes conditions. The individual statistical analysis based on surrogates didn't revealed significant phase synchronization neither for the PSI, nor for the DT, except for the DT in one participant and this in all conditions but the Audio Double condition.

This confirmed that phase synchronization between the hands and the metronome was not present in the various metronomes conditions and that only a frequency relationship was maintained.

Finally a Pearson's correlation analysis indicated that the variability of the juggling pattern increased with the FSI. This was evidenced for BFT (R^2^ = 12%, p = .037), ThVel (R^2^ = 25%, p = .002), and DBTz (R^2^ = 56%, p = 10^−6^). Moreover, we found that the frequency difference (FSI) affected the variability of DBTz in the case of the antiphase audio-tactile metronome (R^2^ = 40%, p = 2*10^−4^) more than in the other conditions (R^2^ = 12% to 28%, .002<p<.051; see Table 3).

**Table 3:** Correlation scores between the vertical distance variability (DBTz) and the relation to the metronomes (FSI).

	Multi. Simp.	Multi. Anti.	Multi. Doub.	Tact. Doub.	Audio. Doub.
R^2^	0.288	0.391	0.209	0.230	0.125
*p*	.010	2*10^−4^	.002	.006	.051

These relate the variability of the vertical distance between left and right consecutive throws (DBTz) and the differences between the frequency of hands movement and the frequency of metronomes (FSI).

## Discussion

Our goal was to examine whether auditory and tactile metronomes, presented together or separately, can influence the behavioral variability of a challenging inter-limb coordination. The question addressed here is that of multimodal integration, and its potentially useful role to convey information from the environment to enhance an ongoing coordination. Recently, rhythmic neuronal mechanisms underlying audio-tactile cross-sensory interactions shed a new light on multimodal integration. It was shown that somatosensory input in auditory cortex reset the phase of ongoing auditory cortical oscillations which subsequently enhanced the auditory response [80]. Whether similar mechanisms may account for the present findings is certainly speculative, but it suggests that rhythmic paradigms involving interlimb coordination may provide a new look to the understanding of multimodal integration phenomena, departing from concepts of simple redundancy [81] or statistical rules [62]. We below discuss how the multiple timing information provided by the audio-tactile metronomes might be integrated to influence the stability of coordination patterns in juggling.

### A tactile metronome can destabilize sensorimotor coordination

We found that a tactile metronome, presented synchronously at the wrists, increased the variability of the velocity at the throws when compared to the baseline variability measured in the absence of a metronome (Tact Doub in Figure 3). This detrimental effect of the tactile metronome on a local component of the juggling coordination, namely individual hand movement, can be interpreted as arising from the well known gating effect that results in tactile suppression, which was also shown in juggling [39]. Alternatively, it can be due to the mismatch between in-phase tactile events and the anti-phase movements of the hands. Please note that the former interpretation calls for a non specific sensory-motor mechanism while the latter emphasize on specific properties of the information specified by the tactile stimuli and its relation to the coordination pattern of the movement of the hand. Further work is required to disentangle, or relate, these two lines of reasoning.

### Advantage of multi-modal over uni-modal conditions

We found that the detrimental effect of the tactile metronome alone could be reduced when presented together with a sound. In particular, we found that the variability of this ball's velocity at throws and its flight duration was lower in the antiphase audio- tactile condition than in the Tact Doub condition (Figure 3 and 4). As the minimization of the variability of the flight duration in skilled jugglers was interpreted as the signature of a global variable of coordination [52], this result suggests that the multimodal stimulation was better integrated within the juggling coordination.

In the present case, each one of the two metronomes brings a distinct tempo, and each tempo is sustained by distinct sensory pathways, allowing for an easier segregation of the two basic rhythms. Deciphering whether such segregation takes place at perceptual, attentional, or more integrated sensori-motor level, requires further work. Taken as an ensemble, these two distinct tempos also define a specific relative frequency and relative phasing between them, and this relative information is the most meaningful to the ongoing coordination. Hence, it seems logical that these properties have collectively contributed to strengthen, or stabilize, the perception-action coordination underlying the relation between the hands and the balls.

As a consequence, the improvement observed when combining audio-tactile stimulations in a multi-frequency multi-modal metronome cannot be exclusively explained by the added contribution of the sound only. Similarly, we cannot argue that the benefit obtained with the audio-tactile conditions was precisely multimodal, because these conditions also specified non negligible relative frequency and a specific relative phasing. We will discuss further the latter below.

We suggest that the audio-tactile stimulations may overcome the tactile detrimental effect, not by acting locally at the level of the throwing movements, but by acting globally onto the ordering the overall pattern of coordination, which requires a tight coupling between the movement of the hands and visual information pick up about the motion of the balls [40], [44], [45].

Another line of thinking is to consider asynchronies in the encoding of visual, auditory and somatosensory cues in the brain. Schroeder and Foxe [82] found evidences in the onset of neural activity in the superior temporal polysensory area for shorter delays following somatosensory stimuli than auditory or visual ones. In the multimodal case, it was suggested that “early-arriving” somatosensory inputs could modulate the local cellular excitability and thus enhance the “later-arriving” auditory input. Such potentiation may explain the improvement observed when combining audio-tactile stimulations in the present experiment, even if only one third of the multimodal stimuli are synchronous because of the 3∶2 coordination involved. Anyway, such suggested multisensory processing is not yet sufficient to explain the improvement found specifically in the case of the multimodal antiphase metronome.

### Parametric coupling of movement to multimodal external events

We examined to what extent the stabilization properties of a double periodic forcing, interpreted formally as a parametric function [59], [65], [83], could generalize to the coordination encountered in a multimodal and multifrequency context. We found a reduction of variability by the double metronome when compared to the simple metronome close to the significance level (Figure 5, p = .051). However when compared to the baseline level of variability taken with respect to the control condition, this stabilization effect did not reach significance (Figure 5). Thus, our results provide only partial support to the idea that the stabilization effect of double periodic forcing extends to a multimodal and multifrequency context. This generalization requires further validation, which would confirm the assumption that the underlying parametric coupling is acting at an abstract level and is amodal [83].

### Phasing of the metronomes matters

We found that, for ball flight duration and vertical distance between throws, variability in the Multi Simp condition was higher than in the Multi Anti condition (Figure 5). Let us recall that these two metronomes differed only by the relative phasing between the tactile events. This specific phasing of the tactile metronome matched the antiphase pattern of hands movement required to perform the 3 balls cascade trick, and is likely to be one important contribution to a more regular behavior. This is a first indication that the phasing of the stimulation (whether multimodal or not) is of high importance for the juggler. A second indication is that the antiphase metronome was the only one that effectively helped the juggler to be more stable. More precisely, matching the phasing of the tactile stimulations with the actual phasing of the movement of the hands reduced the variation of distance between successive projection points of left and right hands (i.e., Multi Anti is lower than zero in Figure 5), presumably by favoring a specific instance of the so-called anchoring phenomena [55]–[58].

Taken together, these two indications denote the key role of the relative phasing in the effective integration of external stimuli within an ongoing coordination pattern: the degree to which information can be useful is the degree to which it can be integrated within the intrinsic behavioral dynamics [54]. Furthermore, a multimodal improvement of movement coordination may be conditioned by a matching of the structure of the coordination, as suggested by the advantage of providing non coincident tactile stimulation corresponding to the anti- phase movement of the hands.

### The mediating role of synchronization to the stimuli

The variability of the juggling coordination covaried with the difference of frequency between hands movement and the metronomes. These frequency gaps varied because some participants accelerated or slowed down during the course of the experiments. This suggests that synchronization favors the integration of the parameters specified by the metronomes to enhance spatial and temporal coordination. Participants were only at an intermediate level in juggling skills, and it was difficult for them to match their movement to the stimuli, hence only weak frequency locking was established. This explained why we didn't find systematic phase synchronization epochs during a trial, only a loose frequency concordance between metronome and hand indicative of weak entrainment, which however was sufficient to pass on the information about the periodic parameters to affect the coordination.

### Conclusion

Much work has gone toward identifying robust rules for multisensory combination and integration [3], [5], [6]. Firstly, when compared to uni-sensory events, two or more sensory cues from distinct modalities with spatial or temporal congruency can elicit a better spatial detection, orientation behavior, or shorter reaction time. Secondly, departure of multimodal environments from spatial contiguity and/or synchrony fails to improve such elementary behaviors when compared to unimodal performances, but can facilitate the execution of other type of tasks, for instance the identification of a temporal gap between events [15]. These two cross- modal phenomena can be tentatively classified respectively as integration, to express a degree of fusion between functional units (e.g., percepts, actions), and as segregation, to indicate a degree of separation between units [84], [85]. One may assume that, depending on the coordination pattern required by the task, integration or segregation may be sought using multimodal stimuli. The multimodal and multifrequency metronomes specified also a timing relation between the two main components of the coordination problem of juggling, because it associated tactile events matching the tempo of the hands and sound events matching the tempo of the balls. Therefore, the present study may be seen as an attempt to use cross-modal stimuli to segregate two frequencies putatively relevant for two components of the coordination, but ultimately to enhance the overall juggling coordination. The latter was presumably determined also by the phasing between the two stimuli in a multimodal pair, associated to antiphase tactile stimuli, despite the weak entrainment of movement to the stimuli.

One specific combination of auditory and tactile metronome decreased of the spatiotemporal variability of the juggler's performance: a sound associated to left and right tactile cues presented antiphase to each other, the latter which corresponded to the temporal pattern of hands movement in the juggling task. Differently we found that tactile cues presented alone increased the behavioral variability. Because audio-tactile events efficiently complemented vision in the regulation of movements in a challenging task and under severe physical constraints, we argue that this class of bimodal combination could, if properly scaled, be used in a wide class of applications to guide and stabilize behavior in the case of efficient or deficient behavior, to accelerate skill acquisition or rehabilitation, and to improve prosthesis. Whether such applications are sought within a limb or between limbs, the coordination has to be carefully analyzed to identify which information, spatial and temporal, should be specified to benefit from a multimodal synergy. Clearly such direction of research could widen the set of laws already established for multisensory integration, namely the laws of inverse effectiveness and coincidence in time and space.

## Supporting Information

Appendix S1 Within participant statistical test for phase synchronization.
(DOC)
