# Supplementary material for: Behavioral Impact of Unisensory and Multisensory Audio-Tactile Events: Pros and Cons for Interlimb Coordination in Juggling
Source: PLoS One. 2012 Feb 27;7(2):e32308. doi: 10.1371/journal.pone.0032308 (PMC3288083; doi:10.1371/journal.pone.0032308)
Supplement: Appendix S1 — Within participant statistical test for phase synchronization. (DOC) [file pone.0032308.s001.doc]

**Appendix S1. Within participant statistical test for phase synchronization**

Phase synchronization of two oscillators means that the phase difference between the two oscillators stays within a given interval, and remains stationary for several cycles. To test for significance we need to determine a threshold value of our measures of phase synchronization, that were synchronization index and dwell time. In the present case, one problem is that the threshold should be calculated individually for each participant, and using a method that allows for a statistical approach. In short, the solution we chose was to compute the threshold as the 95^th^ percentile of the distributions of synchronization index and dwell time, computed from surrogate relative phase time series, to get a threshold value corresponding to a p value of 0.05 for significant synchronization [79].

More precisely, this threshold was calculated for each participant for the both synchronization measures computed from surrogates relative phase. For each condition, the surrogate relative phase was the phase difference between the phase of the hand (taken from the control condition) and a discrete periodic signal corresponding to the stimuli used for the metronomes. The surrogate stimuli had a period equal to the period used for the metronomes for a given participant. To get time series of relative phase and then distributions of synchronization index and dwell time under the null hypothesis of absence of coupling to the stimuli, hence of absence of a synchronization process, we randomized the initial phase of the stimuli. For each subject, a thousand of such surrogate relative phase time series were computed by taking the movement of the hand time series for one trial and by drawing for each time series the initial time shift of the stimuli from a normal distribution centered on zero, such that the first onset of the stimuli fell within the first period of the hand movement. The relative phase, synchronization index and dwell time, were computed exactly the same way as for the metronome conditions. Accordingly we obtained a distribution of a thousand synchronization index and dwell time for each subject. To get a threshold value corresponding to a p value of 0.05 for significant synchronization we took the 95 percentiles of the distributions of the synchronization index and dwell time for a given participant. Because the participant didn’t have any metronome in the control trials, the synchronization index of the surrogate relative phase time series represented the dispersion obtained by chance and only by non specific constrains imposed by idiosyncratic kinematics of the movement of the hand for a given participant, the phase of which was probed at regular times. For instance probing the phase of the hand movement at regular times has by necessity the tendency to select more often the phases of slowly evolving part of the cyclic movement, which can be significant if the movement is of the discrete type. Hence no underlying synchronization process could determine the obtained distribution of the surrogate circular variance of the relative phase. The same rationale can be applied to the dwell time distribution.
